# Histone 4 Lysine 20 Methylation: A Case for Neurodevelopmental Disease

**DOI:** 10.3390/biology8010011

**Published:** 2019-03-03

**Authors:** Rochelle N. Wickramasekara, Holly A. F. Stessman

**Affiliations:** Department of Pharmacology, School of Medicine, Creighton University, Omaha, NE 68178, USA; rochellewickramasekara@creighton.edu

**Keywords:** H4K20, KMT5A, KMT5B, KMT5C, SUV420H, lysine-methylation, neurodevelopment, mutations, epigenetic, histone methylation

## Abstract

Neurogenesis is an elegantly coordinated developmental process that must maintain a careful balance of proliferation and differentiation programs to be compatible with life. Due to the fine-tuning required for these processes, epigenetic mechanisms (e.g., DNA methylation and histone modifications) are employed, in addition to changes in mRNA transcription, to regulate gene expression. The purpose of this review is to highlight what we currently know about histone 4 lysine 20 (H4K20) methylation and its role in the developing brain. Utilizing publicly-available RNA-Sequencing data and published literature, we highlight the versatility of H4K20 methyl modifications in mediating diverse cellular events from gene silencing/chromatin compaction to DNA double-stranded break repair. From large-scale human DNA sequencing studies, we further propose that the lysine methyltransferase gene, *KMT5B* (OMIM: 610881), may fit into a category of epigenetic modifier genes that are critical for typical neurodevelopment, such as *EHMT1* and *ARID1B*, which are associated with Kleefstra syndrome (OMIM: 610253) and Coffin-Siris syndrome (OMIM: 135900), respectively. Based on our current knowledge of the H4K20 methyl modification, we discuss emerging themes and interesting questions on how this histone modification, and particularly KMT5B expression, might impact neurodevelopment along with current challenges and potential avenues for future research.

## 1. Epigenetic Methylation at H4K20 

**Figure 1:**
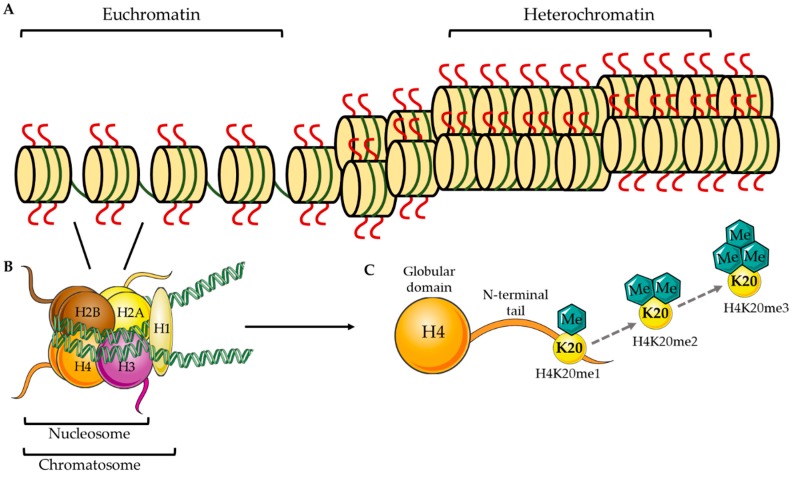
Schematic representation of chromatin organization and H4K20 methylation. (**A**) Models for accessible (euchromatin) vs. condensed (heterochromatin) nucleosomal structures. (**B**) Two each of the histone proteins, H2A, H2B, H3, and H4, come together to form a histone octamer, which is bound by ~1.65 turns of DNA to form the nucleosome. The nucleosome bound by the H1 linker histone forms the chromatosome, which is further compacted to form chromosomes. (**C**) Histone protein 4 has a globular head domain and an N-terminal tail, of which the lysine (K) 20 residue can be mono, di, or tri-methylated.

In eukaryotic organisms, hereditary information is encoded in long strands of DNA that must be compacted and organized in the cell nucleus. This process is accomplished by the wrapping of DNA around histone proteins forming dense nucleosome complexes, collectively termed *chromatin* (Figure 1A). DNA wraps ~1.65 times [1] around an octamer of histone proteins consisting of two copies each of the H2A, H2B, H3, and H4 histone proteins forming a nucleosome (Figure 1B). A nucleosome unit bound by an H1 linker histone forms the chromatosome (Figure 1B), which is further compacted and coiled tightly to form chromosomes [1]. Histone proteins consist of a globular head domain and an N-terminal tail that protrudes from the nucleosomal structure (Figure 1C). These N-terminal tails are permissive to modifications, such as acetylation, methylation, phosphorylation, and ubiquitination, which can translate into more relaxed DNA (euchromatin) that is permissive to transcriptional machinery or more condensed DNA (heterochromatin)—where transcriptional machinery has limited access [2] (Figure 1A). It is hypothesized that these modifications encode a histone code, where, depending on the modification, can be interpreted as various readouts (e.g., gene activation vs. gene silencing or cell proliferation vs. cell differentiation). Further, complexities of this histone code are still being identified; whereas one modification may act independently in one context, the same modification may act together with other modifications in a second context to dictate other cellular events [2,3]. Not surprisingly, histone modifications have emerged as powerful regulators of cell cycle progression, DNA replication, DNA damage repair, lineage specification, and chromosomal stability [4,5]. 

**Figure 2:**
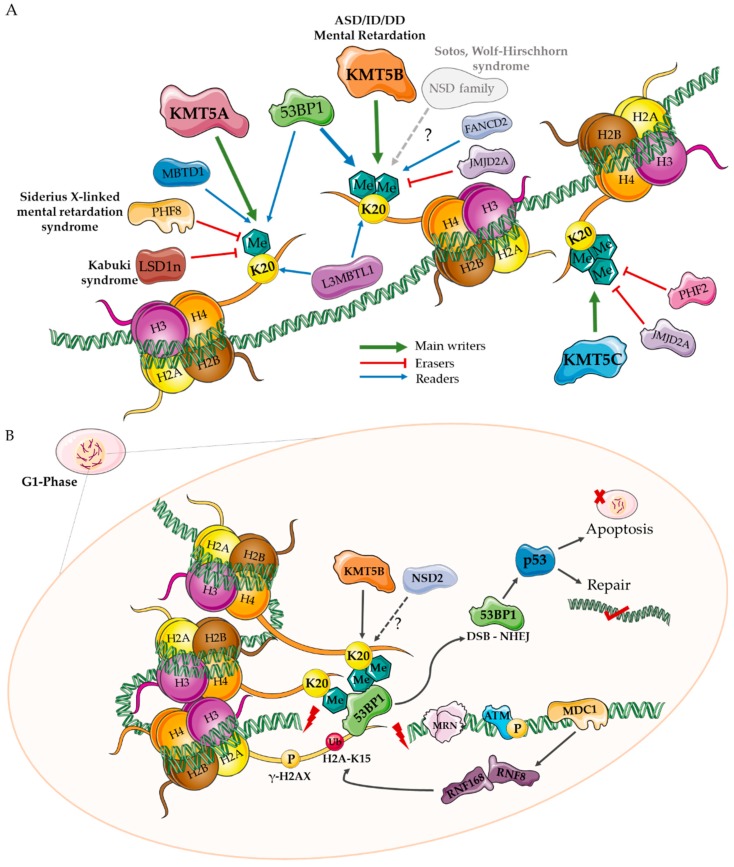
Diagram of major H4K20-related proteins and their proposed role in DNA double stranded break (DSB) repair. (**A**) Lysine methyl transferase (KMT) 5A, KMT5B, and KMT5C are the three main enzymes involved in the sequential mono, di, and tri-methylation of H4K20. Other potential H4K20 methyl transferases include the NSD gene family. Lysine demethylase 4A (KDM4A/JMJD2A), lysine-specific histone demethylase 1 neuronal isoform (LSD1n), plant homeodomain finger 8 (PHF8), and PHF2 are demethylases known to act on H4K20. Malignant brain tumor domain-containing proteins (MBTD1, L3MBTL1), Fanconi Anemia Complementation Group D2 (FANCD2), and p53-bindng protein (TP53BP1, 53BP1) are examples of readers of H4K20 methyl marks. Protein TP53BP1 is a checkpoint protein that binds H4K20me1/me2 and other histone modifications that occur in response to DNA damage to facilitate DSB repair via non-homologous end joining (NHEJ). (**B**) The NHEJ pathway is the preferred method of repair during the G1-phase of the cell cycle [11,12]. DNA DSBs (red bolt) are recognized by the MRN complex (MRE11/RAD50/NBS1) binding, which leads to autophosphorylation of ATM kinase and phosphorylation of histone H2AX on S 139 (γ-H2AX). This creates a biding site for mediator of DNA damage checkpoint protein 1 (MDC1), which recruits the E3 ubiquitin ligases, RNF8 and RNF168, to establish polyubiquitination marks at the break sites [4]. These ubiquitination events recruit TP53BP1 and BRCA1 to the damage site. TP53BP1 binds H4K20me1, H4K20me2, and H2AK15 (ubiquitinated by RNF168) and mediates a cascade of events that prevents end resection, signaling NHEJ factors, including p53, to either repair the DNA or execute apoptosis/autophagy [13].

Methyl modifications occur preferentially on lysine (K) residues of histone proteins H3 and H4. While several residues on H3 (K4, K9, K27, K36, and K79) are subjected to methylation (reviewed elsewhere [6]), the main site of this modification on H4 protein is K20 [7,8]. In this report, we focus on H4K20 methylation, which we propose is highly involved in brain development. H4K20 methylation exists in three states that are thought to occur sequentially from a mono (H4K20me1) to di (H4K20me2) to tri-methylated (H4K20me3) state (Figure 1C). While the ultimate goal of H4K20me3 is thought to be chromatin compaction and transcriptional silencing [9], H4K20me1 and H4K20me2 have been shown to have other cellular roles aside from being just a step in the pathway to the H4K20me3 state (described below). Multiple cellular players contribute to the establishment and maintenance of the H4K20 methylation, including methyltransferases (writers), responsible for laying down methyl marks; demethylases (erasers) that remove them; and effector proteins that specifically recognize the methylated site/state (readers) [10]. The major players that act on the H4K20 modification are discussed below and are represented in Figure 2A.

## 2. H4K20 Writers

KMT enzymes responsible for establishing H4K20 methylation marks are part of a large family of proteins that contain a SET domain: Initially named after three *Drosophila melanogaster* proteins (Suppressor of variegation 3-9 (Su(var)3-9), Enhancer of zeste (E(z)), and the homeobox gene regulator Trithorax (Trx)) [14]. Enzymes with a SET domain, methylate lysine residues using S-adenosyl-methionine/homocysteine as a methyl donor [15,16]. 

### 2.1. KMT5A

KMT5A (aliases: SET8, SETD8, and PR-SET7) is exclusively a H4K20 mono-methyl transferase [17,18] with a SET domain-containing active site that is too narrow to accommodate other methylated species [15,19]. Since its identification [17], KMT5A has emerged as a key mediator of chromatin compaction, cell cycle progression [20] and DNA replication (reviewed in [5,21,22]). KMT5A and the H4K20me1 mark are dynamically regulated throughout the cell cycle, a topic which has been reviewed in detail previously [23]. Briefly, during G1 phase, KMT5A is targeted to specific loci, but ubiquitinated by SCF^Skp2^ and degraded by CRL4^cdt2^ [24,25] prior to S-phase, keeping KMT5A and H4K20me1 at low levels. KMT5A increases during G2, where it mono-methylates newly synthesized histones, that are subsequently modified by KMT5B/C. Following chromosomal condensation, closer to anaphase, KMT5A is phosphorylated on serine 29 by cdk1/cyclinB removing KMT5A from mitotic chromosomes. It is then dephosphorylated by Cdc14 phosphatases and degraded by APC^cdh1^-dependent ubiquitination which permits entry back into G1 [22]. While KMT5A has remarkable specificity toward H4K20 and its primary effector function is in mono-methylation, numerous publications have highlighted crucial roles for KMT5A in methylating non-histone targets. For example, KMT5A methylates proliferating cell nuclear antigen (PCNA) protein [26] at K248 [27], an interaction that is important in the CRL4^CDT2^-mediated degradation of KMT5A and in the DNA damage repair process [28]. Other important non-histone KMT5A targets include: Tumor protein P53 (p53), which is methylated at K382 (p53K382me1) [29] and Numb, which is methylated on K158 and K163 [30]. Protein p53 acts as a tumor suppressor by regulating the expression of genes involved in cell cycle arrest, apoptosis, and senescence. Numb forms a tripartite complex with p53 and promotes apoptosis, which is lost in the presence of its KMT5A methylation [21]. Recent evidence highlights the importance of these processes in high-risk neuroblastoma, in which an elevation of KMT5A results in excessive methylation of p53, leading to anti-apoptotic tumor behavior [31]. 

### 2.2. KMT5B and KMT5C

The enzymes KMT5B (aliases: SUV420H1, SUV4-20H1) and KMT5C (aliases: SUV420H2, SUV4-20H2) are highly homologous and have been investigated together in many studies [9,32]. All KMT5B and KMT5C isoforms share: (1) A catalytic SET-domain, (2) a unique N-domain compared to other SET domain-containing proteins, and (3) a Zn-binding post-SET domain [33]. KMT5B and KMT5C are ~3 times less catalytically active on unmodified H4 than H4K20me1, and KMT5A is 250-fold more efficient at monomethylating H4K20 than KMT5B and KMT5C [33]. These observations highlight the specificity of each enzyme and perhaps different cellular roles. 

KMT5B/C is enriched in peri-centric heterochromatic regions and is typically a mark of silenced heterochromatin [9]. Investigations looking at loss of both KMT5B and KMT5C highlight a global transition to the H4K20me1 state, defects in cell proliferation, and an inability to maintain stem cell pools [32]. In addition to its role in chromatin compaction (i.e., H4K20me3), H4K20me2 is thought to be involved in the DNA DSB repair pathway; KMT5B/C-deficient cells show increased sensitivity to DNA damage and impairments in lineage programs that require DSB repair (immunoglobulin class-switching and VDJ-recombination) [32]. KMT5B/C is linked to the DSB repair process via its main methyl modification, H4K20me2, to which TP53BP1 binds via its tandem tudor domains [34]. Recruited via the NHEJ protein Ku70, KMT5A establishes the H4K20me1 mark at sites of DSBs, which facilitates the recruitment of KMT5B/C to lay down the H4K20me2 mark necessary for TP53BP1 binding [35]. This process seems to be a pivotal determinant of a cell’s decision to repair or die [34]. TP53BP1 is crucial in a cell’s decision to use homologous recombination (HR) or NHEJ to repair DSBs, where the former is mediated by BRCA1 and the latter by TP53BP1 [34,36]. In non-replicating cells, H4K20me2 is more readily available for TP53BP1, which antagonizes BRCA1 binding and consequent HR repair. Conversely, during replication, the laying down of new histones dilutes H4K20me2 marks, favoring BRCA1-mediated repair factors (i.e., HR) [11]. A simplified model for the major players surrounding H4K20 that recognize and orchestrate the repair pathway to resolve DSBs are represented in Figure 2B [4,11,12,13]. 

### 2.3. NSD Family Writers

The NSD (nuclear receptor SET domain-containing) family of histone methyl transferases consists of three enzymes: NSD1, NSD2 (aliases: MMSET, WHSC1), and NSD3 (alias: WHSC1L1), all of which have been implicated in neurodevelopment and cancer syndromes [37]. Mutations in *NSD1* (OMIM: 606681) are associated with Sotos syndrome, characterized by childhood overgrowth, developmental delay, intellectual disability, muscle hypotonia, and seizures [38]. *NSD2* (OMIM: 602952) is associated with Wolf-Hirschhorn syndrome, characterized by facial dysmorphisms, developmental delay, intellectual disability, muscle hypotonia, and seizures [39]. NSD enzymes have specific methyltransferase activity toward mono and di-methylated H3K36 [37,40], and have been shown to have H4K20me1 and H4K20me3 activity in vitro [41,42,43]. The specificity of the NSD family of methyl transferases to H4K20 is currently unclear [44], as other studies find no NSD affinity for H4K20 [40], a discrepancy that may be explained by the nature of the substrate provided to test enzymatic activity [40]. 

Furthermore, studies suggest that H4K20me2 locally increases upon induction of DSBs and that NSD2 (MMSET), recruited via the interaction of its S102 residue and MDC1, is responsible for this local increase [45]. Other evidence argues that H4K20me2 does not increase in response to DSBs, but the previously established H4K20me2 marks (via KMT5A and KMT5B) are more accessible following H4K16 deacetylation [46]. Further evidence attempting to resolve this discrepancy agrees with the latter, that NSD2 does not regulate H4K20 methylation, is not recruited to DSBs, and is dispensable for TP53BP1 foci formation in response to irradiation [35]. However, it is difficult to eliminate variation among the in vitro systems and methods used to induce DSBs. What is currently unknown is whether NSD proteins (as SET-domain-containing lysine methyltransferase enzymes) are fully redundant in the absence of KMT proteins.

## 3. H4K20 Erasers

The dynamic regulation of H4K20 methylation includes not only the methyl transferases but also demethylases, or “erasers.” These enzymes contain flavin-dependent amineoxidase and α-ketoglutarate dependent Jumonji-C (JmjC) domains for demethylating activity [47]. 

### 3.1. KDM4A

Lysine demethylase 4A (KDM4A; alias: JMJD2A), consisting of tandem hybrid tudor domains, is a histone demethylase that has dual specificity toward H4K20me3 and H3K4me3 [48]. KDM4A also binds H4K20me2 with high affinity and must be ubiquitinated by RNF8 and RNF168 E3 ubiquitin ligases during DNA damage repair for recruitment of TP53BP1 [49]. 

### 3.2. PHF8

A class of demethylases in the JmjC family is characterized by an N-terminal PHD (plant homeodomain zinc finger) domain and a catalytic JmjC domain that recognize methylated lysines [47]. In humans, this family consists of plant homeodomain finger 2 and 8 (PHF2 and PHF8) and KDM7A (alias: KIAA1718) [47]. Of these, PHF2 has demethylase activity toward H4K20me3 [50]; PHF8 has demethylase activity toward H4K20me1 as well as H3K9me1 and H3K9me2 [51]. Truncating mutations in *PHF8* are associated with Siderius X-linked mental retardation with cleft lip/palate [52]. Morpholino knockdown of the *PHF8* ortholog in zebrafish (*phf8*) increases H4K20me1 levels, causes craniofacial abnormalities and apoptosis in the brain and neural tube, and impairs jaw development [51]. Mice deficient in Phf8 (on a mixed 129/B6 background) show no gross developmental defects or cognitive defects but show resiliency to stress-induced anxiety and depression-like behaviors through the regulation of serotonin receptors in the prefrontal cortex [53]. In another Phf8 knockout mouse model (C57BL6/J backcross, N5), animals were found to have learning and memory impairments, compromised long-term potentiation, and increased basal synaptic transmission in the hippocampus [54]. Data suggest that Phf8 is involved in the suppression of mTOR signaling [54], a key pathway in both normal and abnormal development (i.e., cancer). Moreover, differences between Phf8 knockout mouse models highlight the importance of controlling both the genetic background and the environment in epigenetic studies of development. 

### 3.3. LSD1/KDM1A

Lysine-specific histone demethylase 1A (KDM1A; alias: LSD1) is primarily described as a H3K4me1 and me2 demethylase implicated in phenotypes of cognitive impairment that resemble Kabuki syndrome (OMIM: 147920) [55,56,57]. However, a brain-specific isoform of LSD1 [58], termed LSD1n, has been shown to have additional H4K20 demethylase activity in vitro and in vivo [59]. Global H4K20me1 levels are increased in Lsd1n-deficient neurons, mainly in transcribed coding regions of neural-activity-related genes. Lsd1n-deficient neurons have increased RNA polymerase II pausing, indicating a role for Lsd1n in transcriptional elongation. Brain-specific Lsd1n knockout mice show defective spatial learning and memory [59]. While LSD1n-mediated brain functions could be due to retained H3K4me2 or H4K20me1 demethylase activity, the evolution of a neuron-specific demethylase isoform may provide better transcriptional control over genes in response to plasticity events [60]. 

## 4. H4K20 Readers

The mono, di, and tri-methyl marks are dynamically read by specialized reader/effector proteins that bind methyl-lysine residues via motifs such as chromo, tudor, MBT, WD40, BAH, ADD, ankyrin, PHD, and zn-CW [10].

### 4.1. MBT, L3MBTL1

Malignant brain tumor D1 (MBTD1) binds H4K20me1 and plays a role in mouse oocyte maturation, TP53BP1-mediated DSB repair, checkpoint activation (reduced cyclin B1, cdc2), and chromosome alignment during mitosis [61]. Lethal (3) Malignant Brain Tumor L (3) (L3MBTL1) is a H4K20me1 and me2 reader [62] involved in compacting nucleosomes [63]. In response to DNA DSBs, ATM-mediated phosphorylation of γH2AX recruits MDC1 and RNF ubiquitination proteins [64]. RNF8-mediated ubiquitination recruits the ATPase protein VCP to the break, which releases H4K20me2-bound L3MBTL1 to facilitate the binding of TP53BP1 (L3MBTL1 has higher affinity for H4K20me2 than TP53BP1) [64]. While the exact neuronal functions of L3MBTL1 need further investigation, this protein is highly expressed in the mature brain, and mice deficient in L3mbtl1 have decreased anxiety and depressive-like phenotypes in various mood-related behavioral tests [65]. 

### 4.2. TP53BP1 

The cell cycle checkpoint protein, p53-binding protein 1 (TP53BP1), is an important reader of H4K20me states as a co-activator of p53, a tumor suppressor that is rapidly activated in response to DNA damage and cellular stress [66], as described above (Figure 2B). The H4K20-TP53BP1 DSB repair pathways have been reviewed previously [13,67,68]. 

### 4.3. FANCD2

Fanconi Anemia Complementation Group D2 (FANCD2; OMIM: 613984), recently identified as a H4K20me2 reader, is associated with the FA/BRCA DNA repair pathway, which promotes HR over NHEJ [69]. FANCD2 acetylates H4K16, which prevents TP53BP1 binding to its docking site, H4K20me2, thus impairing DNA end resection and HR repair [69]. Disruption of the FA-HR pathway results in Fanconi anemia, which is associated with a predisposition to cancer [70]. 

## 5. A Role for KMT Enzymes in Neurodevelopment?

H4K20 methylation has been pathologically implicated in neurodevelopmental disorders (NDDs); however, this has come primarily from demethylase enzymes and the NSD family of methyltransferases, which target multiple histone lysine residues [71]. While many of these disorders have been described in the clinical literature, there is a clear gap in knowledge for the H4K20 methyl writer KMT family of enzymes.

### 5.1. Genetic Evidence for KMT Genes

Among the KMT H4K20 methyl writer family, *KMT5B* (*SUV420H1*) has been most implicated in human phenotypes through multiple independent sequencing studies. While initially highlighted using computational modeling in 2015 [72], two large-scale sequencing publications in 2017 provided additional evidence that *KMT5B* harbors more potentially pathogenic de novo mutations in individuals with NDDs than would be expected by chance under multiple statistical models [73,74]. Exome sequencing of individuals with developmental disorders (n = 4293 [73]) found that only one individual carried a de novo synonymous variant in *KMT5C* and no de novo mutations in *KMT5A* [73,74]. It is important to note, however, that these sequencing studies excluded individuals with known syndromic forms of NDD (e.g., PHF8, KDM1A, NSD1, and NSD2) and were interested primarily in de novo events from simplex families (no family history of disease) [73]. De novo events, although arguably easier to characterize, are more rare than inherited events, [75], suggesting that additional H4K20-acting genes may be significantly associated with NDD, but remain underpowered for detection in current datasets. This hypothesis is supported by the fact that de novo variants have been identified among individuals with NDD for many of the other writers, erasers, and readers of the H4K20 mark (Supplementary Table S1). Further, analysis of the sex chromosomes is difficult and genes that reside there (e.g., *PHF8*) with an X-linked inheritance pattern [52,76] are often difficult to detect (i.e., unaffected carrier mothers with affected sons) and are filtered out of de novo studies as inherited. Finally, the Stessman et al. study that initially identified *KMT5B* as an NDD-associated gene was a targeted sequencing study that did not include any of the other H4K20-associated genes [74].

**Figure 3:**
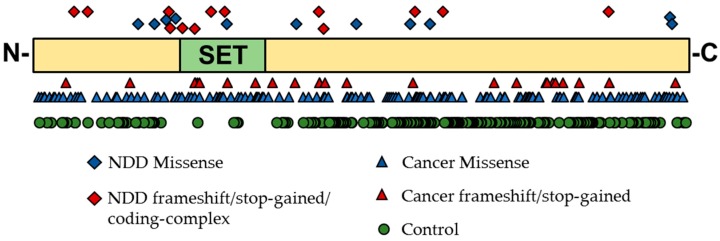
Schematic of the full-length KMT5B protein with its main annotated functional domain, the SET domain. Data points represent single nucleotide variants (SNVs) identified within NDD [73,77,79,80], cancer [81], or control (missense or in-frame deletions) [78] populations. Only SNVs falling within the coding sequence are depicted (i.e., intronic, splice-blocking, and synonymous mutations are excluded). While NDD and cancer variants span the full protein and cluster near the SET domain (likely disrupting gene function), there is a clear lack of mutations near the SET domain among control individuals, indicating that protein altering variants that affect the expression of the SET domain are more-likely associated with disease phenotypes.

To date, we have identified 86 individuals from the literature, the DECIPHER database, or our own studies carrying coding variants in the *KMT* gene family (SNVs and copy number variants (CNVs); Supplementary Table S2 and S3) [73,77,78,79]. This includes 17 CNVs and one missense mutation in *KMT5A*; 11 CNVs, 24 unique missense/frameshift/stop-gained/coding-complex SNVs (Figure 3: NDD data points on top), and eight intronic/3’-UTR/splice-donor SNVs in *KMT5B*; and 23 CNVs and two synonymous/intronic mutations in *KMT5C*. From the ExAC control exomes [80], it is apparent that LoF variants in *KMT5A* and *KMT5B* are highly constrained in typical individuals, whereas variation in *KMT5C* is tolerated (Table 1). Compared to the known disease-associated NSD family of H4K20 writers, *KMT5A* and *KMT5B* would be expected to be equally intolerant to loss-of-function mutations and only slightly more tolerant to missense changes; yet, overall, even some missense changes would be expected to affect protein function (Supplementary Table S4). Interestingly, more synonymous variants were identified in *KMT5C* among controls than would be expected by chance (Table 1). While this does not conclusively rule out *KMT5C* variation as a multigenic risk factor for clinical phenotypes, it could suggest that this is a genetic hotspot for variation.

Variant class scores (z and pLI) taken from the ExAC Browser (http://exac.broadinstitute.org/) representing 60,706 unrelated individuals sequenced as part of various disease-specific and population genetic studies [78]. Associated disorder annotations taken from Online Mendelian Inheritance of Man (OMIM). MR, AD: Mental Retardation, Autosomal Dominant (OMIM nomenclature). Bold values are considered to be constrained.

Most of the 43 individuals carrying *KMT5B* variants have a primary diagnosis of intellectual disability (ID), autism spectrum disorder (ASD), or developmental delay (DD) (Supplementary Table S3). Based on available phenotypic information for only individuals carrying SNVs in *KMT5B* (as CNVs often affect multiple genes, reducing specificity) [74,77,80], prominent NDD phenotypes include: ASD (9/24 = 41%), ID (15/22 = 68%), speech/language delay (7/16 = 43%), motor phenotypes (6/16 = 38%), seizures (4/16 = 25%), and brain abnormalities (6/16 = 38%) (Supplementary Table S5). Denominators varied based on the completeness of phenotypic workups. Brain abnormalities included: Macrocephaly, hydrocephalus, hypoplasia of the corpus callosum, enlargement of ventricles, and differences in size of structures. Motor phenotypes included: Motor delay, motor coordination disorder, delayed gross/fine motor development, and muscle hypotonia. Other notable phenotypes included: Hypermobility of the joints, sleep problems, and dysmorphisms [77,80] (Supplementary Tables S3 and S5). 

### 5.2. Gene Expression Evidence for KMT Genes

Brain-specific functional details and expression patterns of H4K20 methylation and the KMT enzymes (KMT5A, KMT5B, and KMT5C) are currently not well understood, but available expression datasets and model systems give us some leads.

**Figure 4:**
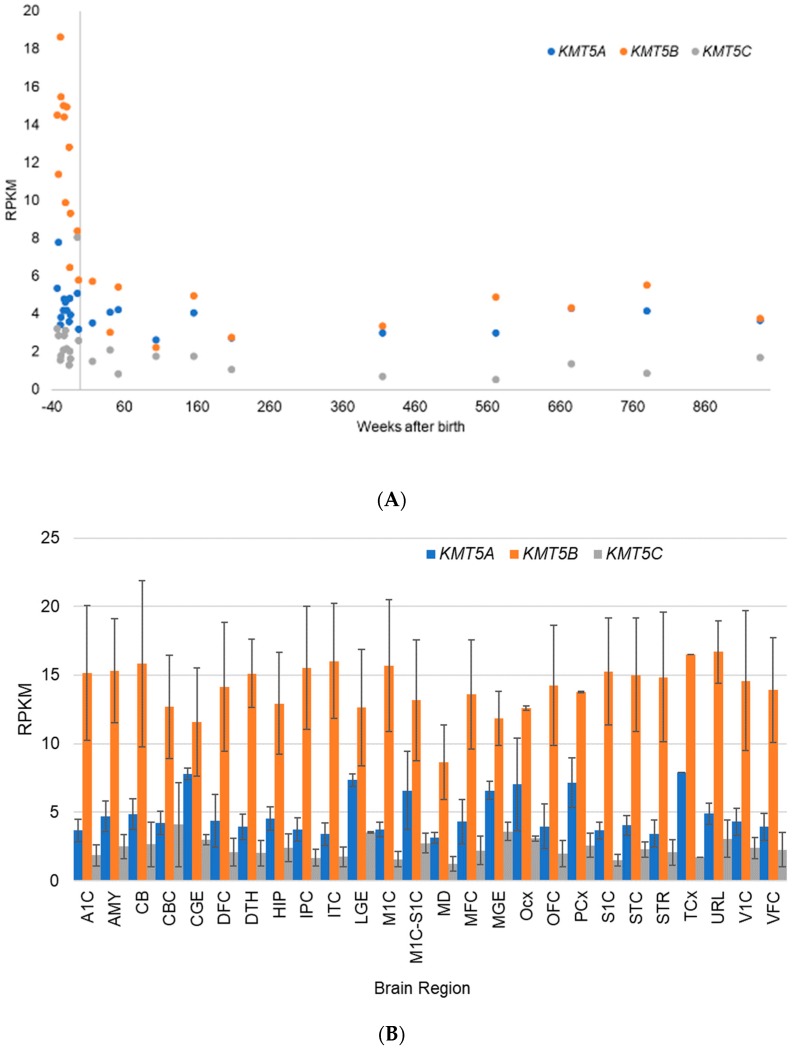
Developmental transcriptome data for the human *KMT* genes from the Allen BrainSpan Atlas [82]. (**A**) Scatterplot shows average expression across all brains regions by age from conception (−40 weeks) to 18 years old (936 weeks); and (**B**) bar graph shows average expression by brain region for data points from all individuals before birth (i.e., week 0). As defined by the Allen BrainSpan Atlas: A1C: primary auditory cortex (core); AMY: amygdaloid complex; CB: cerebellum; CBC: cerebellar cortex; CGE: caudal ganglionic eminence; DFC: dorsolateral prefrontal cortex; DTH: dorsal thalamus; HIP: hippocampus (hippocampal formation); IPC: posteroventral (inferior) parietal cortex; ITC: inferolateral temporal cortex (area TEv, area 20); LGE: lateral ganglionic eminence; M1C: primary motor cortex (area M1, area 4); M1C-S1C: primary motor-sensory cortex (samples); MD: mediodorsal nucleus of thalamus; MFC: anterior (rostral) cingulate (medial prefrontal) cortex; MGE: medial ganglionic eminence; Ocx: occipital neocortex; OFC: orbital frontal cortex; PCx: parietal neocortex; S1C: primary somatosensory cortex (area S1, areas 3,1,2); STC: posterior (caudal) superior temporal cortex (area 22c); STR: striatum; TCx: temporal neocortex (n = 1); URL: upper (rostral) rhombic lip; V1C: primary visual cortex (striate cortex, area V1/17); and VFC: ventrolateral prefrontal cortex. Error bars represent the standard deviation of the mean.

The Allen BrainSpan Atlas is a publicly available resource for identifying transcriptional mechanisms involved in human brain development [82]. The available RNA-Sequencing dataset represents up to 26 brain regions that have been isolated and preserved on a standard protocol from 42 “normal” human donors ranging in age from eight post-conception weeks (pcw) to 40 years. All RNA-Seq reads were mapped to the human reference genome, and following normalization, are reported in the commonly used units of reads per kilobase of transcript per million mapped reads (RPKM). All documentation related to this dataset is publicly available (http://help.brain-map.org/display/devhumanbrain/Documentation). These gene expression data show that *KMT5B* and *KMT5C* transcripts are most highly expressed prenatally, with a decrease to steady state levels after birth ([82], Figure 4A). The contrast in gene expression before and after birth is the most dramatic for *KMT5B* (Figure 4B). These data further suggest that *KMT5B* expression is positively correlated with neurogenesis (Supplementary Figure S1A), as the highest levels of *KMT5B* expression occur up to 20 weeks post-conception (Supplementary Figure S1B), dropping steadily until birth (Supplementary Figure S1C), after which a steady state is maintained (Supplementary Figure S1D–E). Postnatal expression of *KMT5B* is approximately equal to *KMT5A*; *KMT5C* maintains constitutive low-level expression across all timepoints and brain regions (Supplementary Figure S1B–E).

In situ hybridization data from the Allen Mouse Brain Atlas show that *Kmt5a*, *Kmt5b*, and *Kmt5c* are also expressed in the young adult mouse brain [83] (P56; approximate human equivalent = 16–19 years of age [84]) in unique patterns (Supplementary Figures S2–S4) [83]. Highest expression for *Kmt5b* is shown in the hippocampus, specifically in the dentate gyrus and field CA3 pyramidal layer (Supplemental Figure S3). Interestingly, the hippocampus is one of few brain tissues that undergoes neurogenesis through adulthood [85]. Spatial and temporal differences in H4K20me1 and me3 are also evident in the E9.5 developing mouse neuroepithelium, where H4K20me1 is highly expressed in medially located luminal cells that are dense in proliferating neuroblasts, and H4K20me3 is highly expressed in laterally located cells that are undergoing differentiation [86]. Levels of H4K20me3 are virtually absent from rapidly proliferating neuroblasts, but within a day become highly enriched for H4K20me3 in ventrolateral aspects of the embryo, an area known to give rise to mature spinal motor neurons and dorsal root ganglia [86]. Thus, H4K20me1 appears to be permissive of proliferation and H4K20me3 is involved in maturation and differentiation in the process of neurulation. While a systematic analysis of H4K20 mono, di, and tri-methylation in the human brain is not yet available, H4K20me3 levels increase around many of the promoter regions of glutamate receptor genes in the adult human cerebellar cortex compared to the fetal cerebellar cortex [87]. It is possible that H4K20 methylation contributes to the maturation of the cerebellar cortex by silencing excitatory glutamatergic transmission genes.

### 5.3. Model Systems Evidence for KMT Gene Involvement in Neurodevelopment

Loss of PR-Set7 (*D. melanogaster* homolog for *KMT5A*) in *D. melanogaster* larval brains causes disorganization of highly proliferative regions of the optic lobes. Based on the severe chromosomal condensation defects seen in mutant flies [88], KMT5A is likely to have a crucial role in early brain development when neuronal migration takes place. While flies lacking PR-Set7 survive until the larval/pupal transition, *Kmt5a* homozygous-null mice can only be recovered at 2.5 days post-conception (cleavage stage) [89]. Embryonic stem cells derived from these embryos lack all three methylation marks (H4K20me1, H4K20me2, and H4K20me3), have major chromosome de-condensation, DSB accumulation, delay in S-phase cycling, and ultimately arrest at G2/M [89]. These data highlight the importance of KMT5A during development and lead us to hypothesize that the paucity of mutations observed in humans (control and clinical studies alike) is likely due to embryonic lethality. 

Clues to the roles of KMT5B and KMT5C in the developing brain come from zebrafish, amphibian, rodent, and primate model systems. In situ hybridization in zebrafish shows that zebrafish orthologs *SETD8a* and *SETD8b* (*Kmt5a*), *suv420h1* (*Kmt5b*), and *suv420h2* (*Kmt5c*) are ubiquitously expressed before the onset of zygote gene expression, with higher expression in the head region [90,91]. Morpholino knockdown of *KMT5B*/*C* orthologs in *Xenopus* embryos results in defective eye and melanocyte differentiation, reduced cell proliferation, and increased apoptosis during development, likely resulting from impairing transition from a pluripotent state to a differentiating state, highlighting a role for KMT5B/C enzymes in neural fate determination [92]. Consistent with the role of KMT5B/C enzymes in cell cycle regulation, the primate subventricular zone (SVZ)—an adult neurogenic niche rich in progenitor cell types [85]—is enriched for H4K20me3-positive GFAP, nestin, and DCX-positive neural stem and progenitor cells that contribute to lifelong neurogenesis [93]. Conditional deletion of both Kmt5b and Kmt5c in the SVZ of the adult mouse brain decreases the number of proliferating S-phase cells after five days, with no effect on mitosis [93], yet increases the number of proliferating cells in the sub-granular zone/dentate gyrus neurogenic niche at 46 days [94]. The reason for these spatiotemporal differences in proliferation in Kmt5b/c knockout mice is unclear. Moreover, *Kmt5b*-null mice (deletion of the entire SET domain) show perinatal lethality, while *Kmt5c* null mice have no apparent phenotype [32]. Deletion of both *Kmt5b* and *Kmt5c* recapitulates the perinatal lethality seen in *Kmt5b*-null mice, suggesting that there is a profound need for Kmt5b during development [32].

While many of these studies are circumstantial at implicating H4K20 methylation in various neuropathological states, they give credence to the ability of H4K20me1 and me2 to modulate juvenile-to-adult plasticity states in the brain and the role of H4K20me3 locking in neural phenotypes. 

## 6. Discussion 

**Figure 5:**
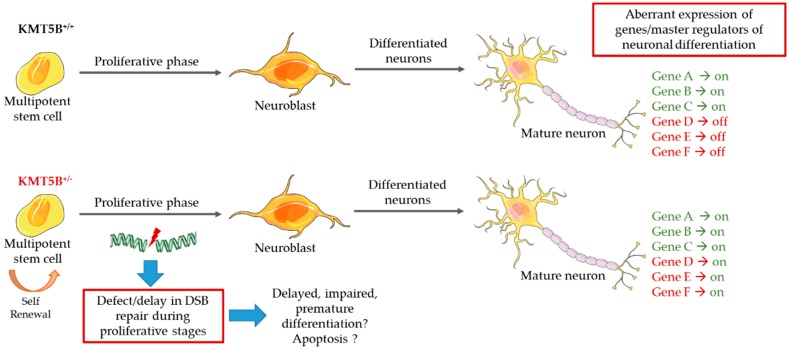
Proposed dual model for KMT5B haploinsufficiency. We propose that KMT5B loss-of-function could result in (1) aberrant gene expression changes and/or (2) defective DSB repair, leading to defects in proliferation and differentiation of neural cells responsible for governing NDD phenotypes.

Epigenetic mechanisms of gene expression provide an additional level of fine-tuning that has been linked to developmental phenotypes. A review of the current literature highlights the importance of H4K20 methylation in normal developmental processes, and we would argue, in the brain. Currently available data suggest multiple roles for KMT5B and the H4K20me2 mark, yet there is an absence of research to definitively support this. This is likely due to the multiple roles this gene plays in the cell. On the one hand, it is the classical role of gene repression often associated with H4K20 methylation. In this role, KMT5A performs H4K20me1, KMT5B H4K20me2, and KMT5C H4K20me3, ultimately resulting in DNA compaction and gene silencing. One hypothesis for KMT5B loss in this case is decreased H4K20me3, more open chromatin/gene expression, and less differentiation, which requires quiescence (Figure 5). This is supported by in vitro work in human primary fibroblasts [95]. On the other hand, KMT5B-H4K20me2 may be a critical determinant of the cells ability to resolve DSBs. As a versatile checkpoint protein, TP53BP1 recruitment and binding depends on other acetyl, methyl, and ubiquitin histone post-translational modifications [96]. Moreover, TP53BP1 can bind the DNA DSB mark γ-H2AX directly without the requirement for H4K20me2 [36]. With the vast number of proteins involved in the repair process, it is unlikely that a lack of H4K20me-TP53BP1 binding would sufficiently impair this process; however, truncating/loss-of-function or missense mutations in *KMT5B* that decrease activity or potential gain-of-function missense mutations may have more profound consequences during brain development, when there is a need for rapid cell proliferation and differentiation (Figure 5). Therefore, the KMT5B-H4K20me2-TP53BP1 DSB repair pathway poses interesting questions as to whether a cell, or specifically a neural progenitor, can still effectively repair DSBs in the absence of KMT5B. Is the repair delayed? If DSB repair is defective, will progenitors undergo apoptosis? Can other proteins compensate for KMT5B function in its absence? Is impaired DSB repair the primary KMT5B function driving our patient phenotypes or gene expression changes or a combination of both (Figure 5)? 

The timing of gene expression and known functions of KMT5B suggest a role for this gene in the maintenance of stem cell pools. This is further supported by studies of myoblast differentiation (the skeletal muscle niche) in mice. Actively proliferating myoblasts in primary limb bud mesenchymal cultures and undifferentiated C2C12 muscle cell lines show high H4K20me1 levels [97], which gradually decrease and become enriched in H4K20me3 as they differentiate, indicating a clear switch in the epigenetic landscape from mono-methyl H4K20 to tri-methyl H4K20—a process that must happen sequentially—in response to muscle differentiation [86,98,99]. Subsequently, a crucial role for Kmt5b in muscle stem cell maintenance was identified [100]. Muscle stem cells activate, proliferate, and differentiate into multinucleated myofibers, a process particularly important in response to muscle injury. In mouse striated muscle, Kmt5a and H4K20me1 are increased in proliferating muscle stem cells, while Kmt5b is expressed in quiescent muscle stem cells that are attached to myofibers; Kmt5c is found in myonuclei of differentiated muscle fibers, confirming distinct roles for the three methyl transferases in muscle regeneration and maintenance of “stemness”. Conditional deletion of Kmt5b in muscle tissue depletes the quiescent muscle stem cell population and increases the activated stem cell population, resulting in an inability to regenerate skeletal muscle long-term, following injury [100]. The role of Kmt5b in muscle differentiation has been further verified in cell line and disease models of muscular dystrophy, where mice with even a partial muscle-specific knockout of Kmt5b display centrally nucleated myofibers and necrosis [101]. Further, changes in H4K20 methylation and *KMT5B* expression in human cancers [102,103,104,105] also support a link to maintenance of cell “stemness”, as cancer cells often de-differentiate during transformation to gain motility and the ability to divide at will [106].

Our understanding of the role of epigenetic regulation in neurodevelopment is just beginning. The main support for our argument that KMT5B is involved in neurogenesis through maintenance of the neural stem cell pool comes primarily from the analysis of available human and mouse RNA expression datasets [82,83]. However, gene expression is not necessarily correlated with protein expression. Many groups, including ours, have been severely limited by a lack of specific antibodies that can distinguish between the expression of KMT5A, KMT5B, and KMT5C. While we can robustly detect the H4K20 methylation state (a surrogate used by many as a readout of KMT enzyme activity), this method is insufficient to establish the spatiotemporal specificity of H4K20 writers and erasers and misses the potential non-histone functions of these enzymes completely. We believe that a combination of genetically engineered rodent models for developmental and behavioral assessment combined with in vitro work to pinpoint additional non-histone methylation targets will be required to resolve the cellular function(s) of KMT5B.

Either through the process of gene expression regulation or DSB repair, it is clear that the KMT enzymes are crucial for regulating cell cycle dynamics, proliferation, differentiation, cell fate determination, and specification of lineage programs [32,107]. Lack of precise control over these events has clear consequences, as can be seen in cancer and NDDs. We argue that a systematic investigation of the role of the H4K20 methylation state (including writers, erasers, and readers) in the nervous system, within a carefully-controlled genetic background and environment, will be required to elucidate subtle NDD phenotypes that may be associated with variation in KMT family proteins (Figure 5). Pre-clinical animal models provide a vital resource for investigating the spatial and temporal expression of KMT enzymes during brain development, their role in regulating gene expression changes that are necessary for neuronal proliferation and differentiation, and the role of DSB repair in these processes. Given this level of information, we might better understand the biological role of protein-altering variants in KMT enzymes, provide families with the necessary information to better manage KMT-related conditions (e.g., speech/language therapy and motor skill interventions during critical developmental periods), and develop precision medicine approaches. 

## Figures and Tables

**Table 1:** Constraint metrics by gene and variant class for H4K20 methyltransferases.

Gene (HUGO)	Alias	Synonymous (z)	Missense (z)	LoF (pLI)	Associated Disorder
*KMT5A*	*SETD8*	0.66	2.44	**0.95**	-
*KMT5B*	*SUV420H1*	−0.29	2.71	**1.00**	MR, AD
*KMT5C*	*SUV420H2*	−1.82	1.99	0.69	-

## Supplementary Materials

The following are available online at https://www.mdpi.com/2079-7737/8/1/11/s1, Figure S1: Developmental transcriptome data for the human *KMT* genes from the Allen BrainSpan Atlas, Figure S2: Mouse in situ hybridization (ISH) data for *Kmt5a* (*Setd8*), Figure S3: Mouse ISH data for *Kmt5b* (*Suv420h1*), Figure S4: Mouse ISH data for *Kmt5c* (*Suv420h2*), Table S1: SNVs in other H4K20 methyl writer, eraser, and reader genes, Table S2: CNVs in KMT genes from DECIPHER v9.26, Table S3: SNVs in KMT genes, Table S4: Genetic variation tolerance scores from the Exome Aggregation Consortium by gene, Table S5: Phenotypic summary of available data for KMT gene variant carriers.
