# Supplementary material for: Histone 4 Lysine 20 Methylation: A Case for Neurodevelopmental Disease
Source: Biology (Basel). 2019 Mar 3;8(1):11. doi: 10.3390/biology8010011 (PMC6466304; doi:10.3390/biology8010011)
Supplement: Supplementary file 1 [file biology-08-00011-s001.pdf]

biology

MDPI

Review

Histone 4 Lysine 20 Methylation: A Case for Neurodevelopmental Disease

Rochelle N. Wickramasekara and Holly A. F. Stessman *

Department of Pharmacology, School of Medicine, Creighton University, Omaha, Nebraska, USA; rochellewickramasekara@creighton.edu

* Correspondence: hollystessman@creighton.edu; Tel.: +01-402-280-2255

Supplementary Materials

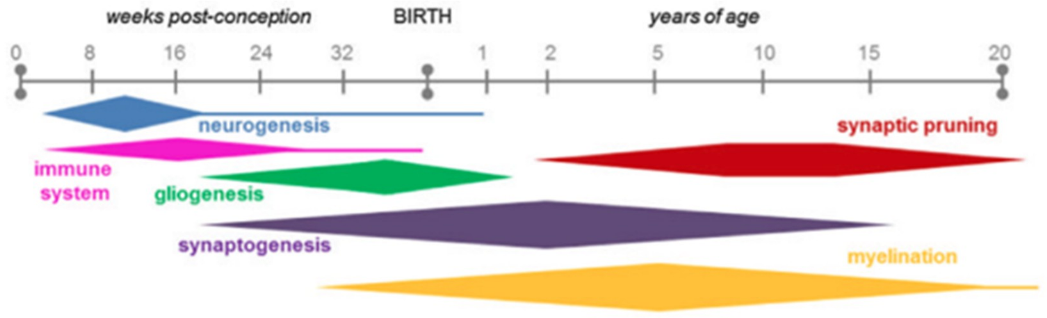

(**A**)

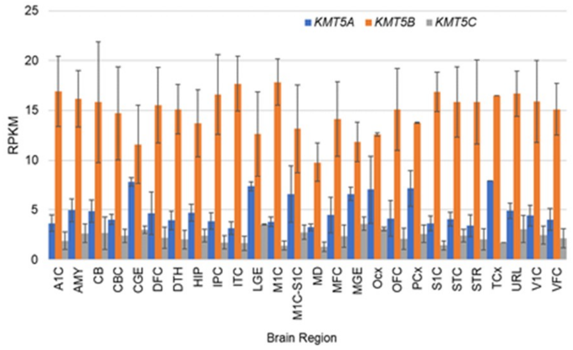

(**B**) Conception to 20 pcw

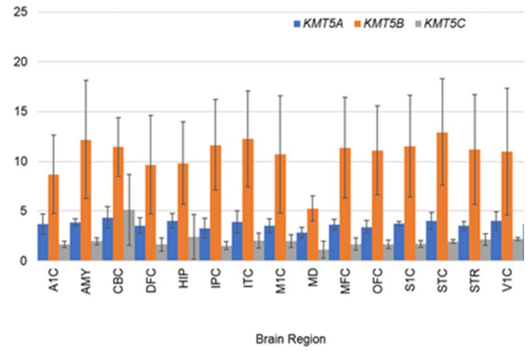

(**C**) 20–40 pcw

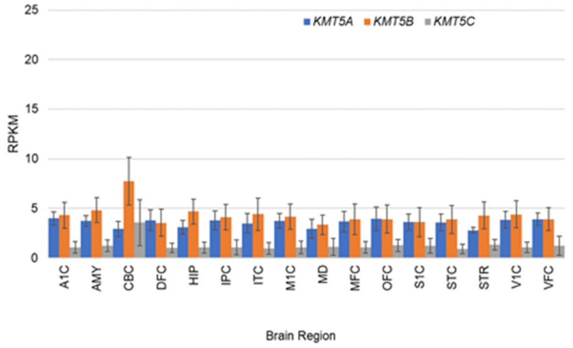

(**D**) Birth to 18 years of age

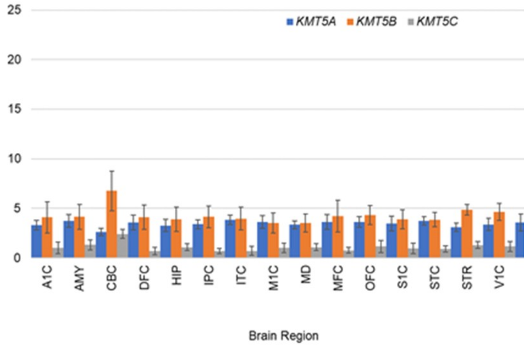

(**E**) > 18 years of age

Figure S1. Developmental transcriptome data for the human *KMT* genes from the Allen BrainSpan Atlas [1]. (**A**) Human neurodevelopmental time course of key processes as modified from Semple *et al*. 2013 [2]. (**B–E**) Bar graph shows average expression by age and brain region for data points from all available individuals as defined by the Allen BrainSpan Atlas [1]: pcw: post-conception weeks;

A1C: primary auditory cortex (core); AMY: amygdaloid complex; CB: cerebellum; CBC: cerebellar cortex; CGE: caudal ganglionic eminence; DFC: dorsolateral prefrontal cortex; DTH: dorsal thalamus; HIP: hippocampus (hippocampal formation); IPC: posteroventral (inferior) parietal cortex; ITC: inferolateral temporal cortex (area TEv, area 20); LGE: lateral ganglionic eminence; M1C: primary motor cortex (area M1, area 4); M1C-S1C: primary motor-sensory cortex (samples); MD: mediodorsal nucleus of thalamus; MFC: anterior (rostral) cingulate (medial prefrontal) cortex; MGE: medial ganglionic eminence; Ocx: occipital neocortex; OFC: orbital frontal cortex; PCx: parietal neocortex; S1C: primary somatosensory cortex (area S1, areas 3,1,2); STC: posterior (caudal) superior temporal cortex (area 22c); STR: striatum; TCx: temporal neocortex (n = 1); URL: upper (rostral) rhombic lip; V1C: primary visual cortex (striate cortex, area V1/17); VFC: ventrolateral prefrontal cortex; RPKM: Reads Per Kilobase of transcript per Million mapped reads. Error bars represent standard deviation of the mean.

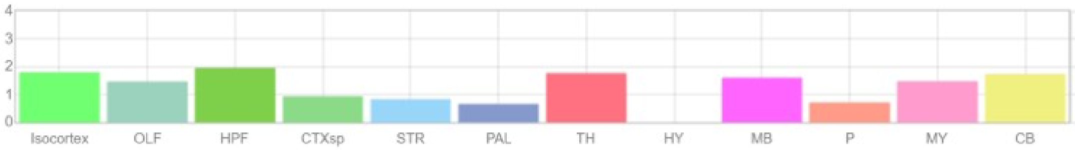

(**A**)

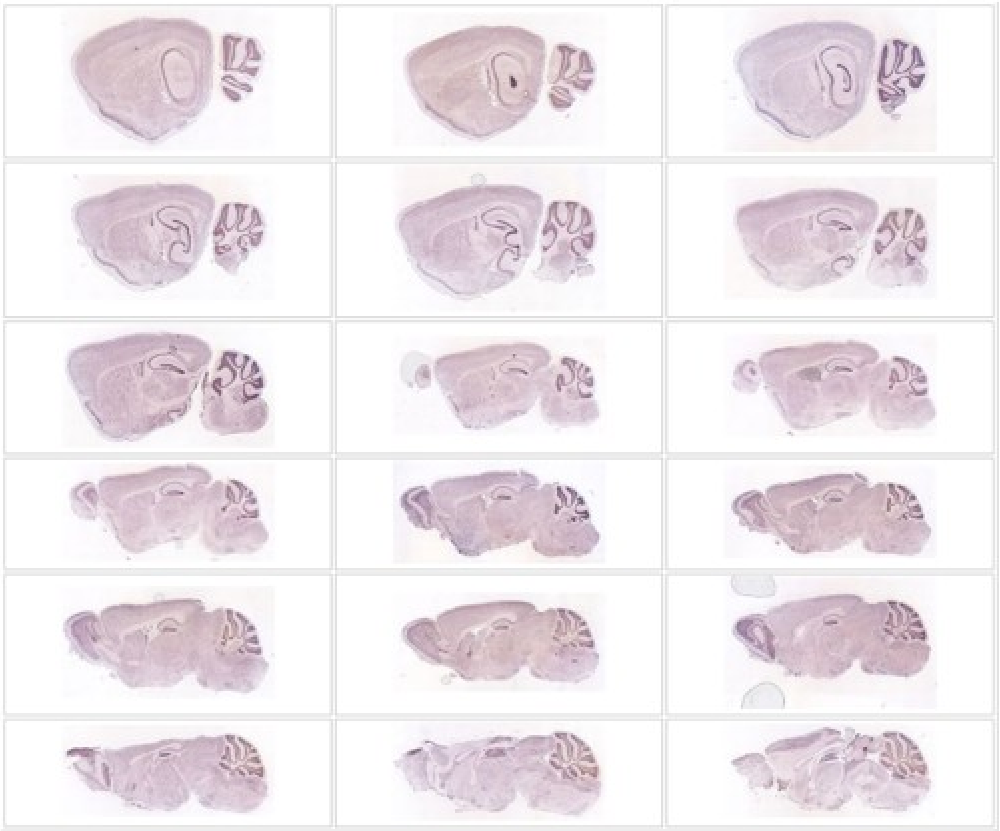

(**B**)

Figure S2. Mouse *in situ* hybridization (ISH) data for *Kmt5a* (*Setd8*). (**A**) Bar graph quantification of (**B**) ISH data by brain region in a P56 C57BL/6J male mouse. For panel (**A**): y-axis: log_2(raw expression value); x-axis: tissue; OLF: Olfactory areas; HPF: Hippocampal formation; CTXsp: Cortical subplate; STR: Striatum; PAL: Pallidum; TH: Thalamus; HY: Hypothalamus; MB: Midbrain; P: Pons; MY: Medulla; CB: Cerebellum. Image credit: Allen Mouse Brain Atlas [3]. An interactive version of these images can be found at this link: http://mouse.brain-map.org/experiment/show/73584831.

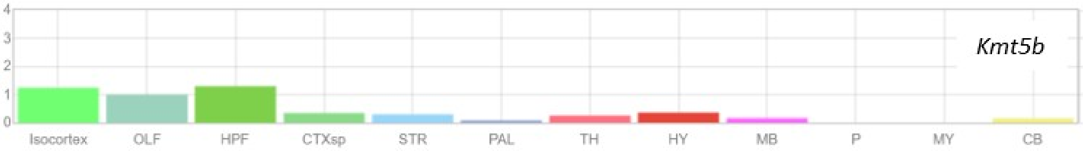

(**A**)

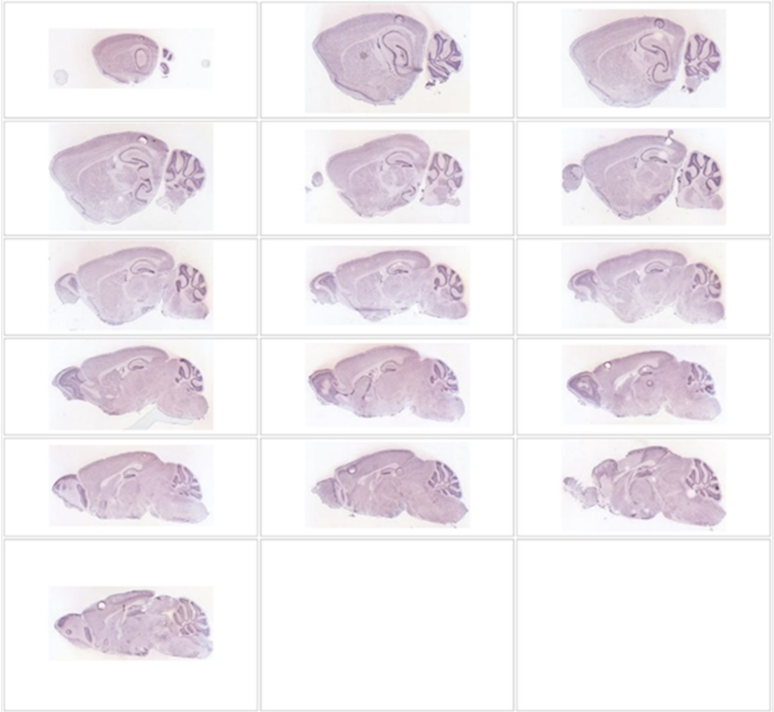

(**B**)

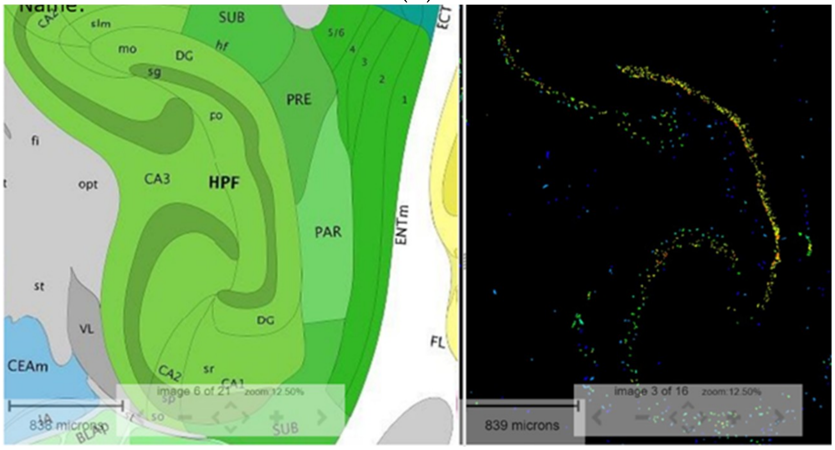

(**C**)

Figure S3. Mouse ISH data for Kmt5b (Suv420h1). (**A**) Bar graph quantification of (**B**) ISH data by brain region in a P56 C57BL/6J male mouse. For panel (**A**): y-axis: log2(raw expression value); x-axis: tissue; OLF: Olfactory areas; HPF: Hippocampal formation; CTXsp: Cortical subplate; STR: Striatum; PAL: Pallidum; TH: Thalamus; HY: Hypothalamus; MB: Midbrain; P: Pons; MY: Medulla; CB: Cerebellum. (**C**) Representative gene expression image for HPF region highlights Kmt5b expression in the dentate gyrus and field CA3, pyramidal layer of the hippocampus. Image credit: Allen Mouse Brain Atlas [3]. An interactive version of these images can be found at this link: http://mouse.brain-map.org/experiment/show/69169427.

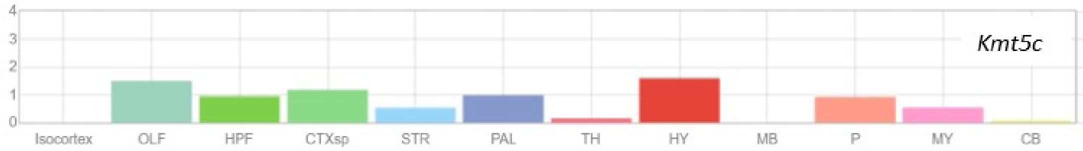

(**A**)

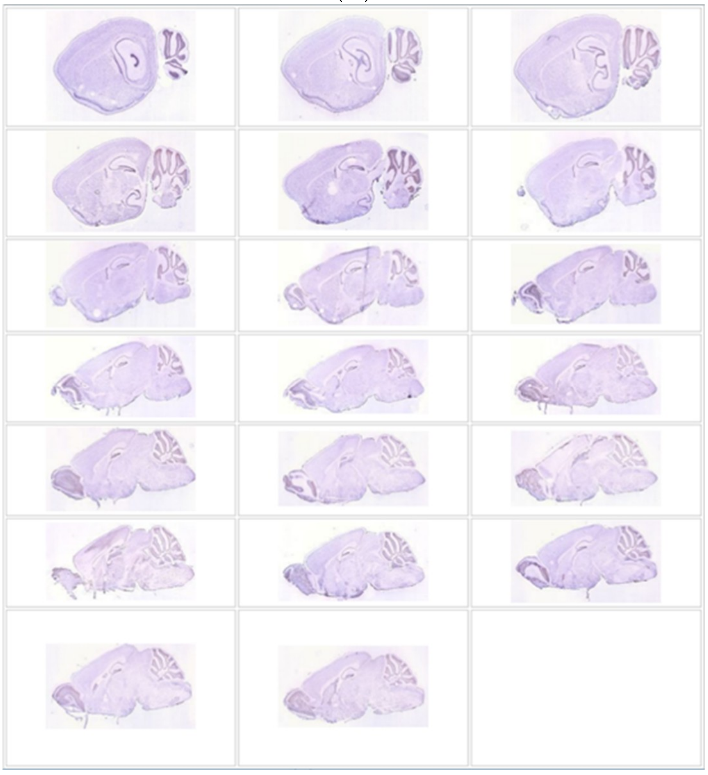

(**B**)

Figure S4. Mouse ISH data for Kmt5c (Suv420h2). (**A**) Bar graph quantification of (**B**) ISH data by brain region in a P56 C57BL/6J male mouse. For panel (**A**): y-axis: log2(raw expression value); x-axis: tissue; OLF: Olfactory areas; HPF: Hippocampal formation; CTXsp: Cortical subplate; STR: Striatum; PAL: Pallidum; TH: Thalamus; HY: Hypothalamus; MB: Midbrain; P: Pons; MY: Medulla; CB: Cerebellum. Image credit: Allen Mouse Brain Atlas [3]. An interactive version of these images can be found at this link: http://mouse.brain-map.org/experiment/show/68148736.

References

1. Miller, J.A.; Ding, S.L.; Sunkin, S.M.; Smith, K.A.; Ng, L.; Szafer, A.; Ebbert, A.; Riley, Z.L.; Royall, J.J.; Aiona, K., et al. Transcriptional landscape of the prenatal human brain. *Nature* **2014**, *508*, 199-206, doi:10.1038/nature13185.
2. Semple, B.D.; Blomgren, K.; Gimlin, K.; Ferriero, D.M.; Noble-Haeusslein, L.J. Brain development in rodents and humans: Identifying benchmarks of maturation and vulnerability to injury across species. *Prog Neurobiol* **2013**, *106-107*, 1-16, doi:10.1016/j.pneurobio.2013.04.001.
3. Lein, E.S.; Hawrylycz, M.J.; Ao, N.; Ayres, M.; Bensinger, A.; Bernard, A.; Boe, A.F.; Boguski, M.S.; Brockway, K.S.; Byrnes, E.J., et al. Genome-wide atlas of gene expression in the adult mouse brain. *Nature* **2007**, *445*, 168-176, doi:10.1038/nature05453.

Table S1. SNVs in other H4K20 methyl writer, eraser, and reader genes.

Gene	Patient	Sex	gDNA position (GRCh37)	cDNA Change	Transcript	Protein Change	Consequence	Pathogenicity	Inheritance / Genotype	Phenotypes	Source
FANCD2	305037	46XY	3:10085544	c.1130A>G	ENST00000287647	p.His377Arg	missense	Uncertain	Maternally inherited (het), constitutive in mother	Autistic behavior; Delayed speech and language development; Thrombocytosis	DECIPHER v9.26
FANCD2	AU4465303	unknown	3:10106444	c.2053G>A	NM_033084.3	p.(G685R)	missense		unknown	autism	PMID:28263302
FANCD2	305037	46XY	3:10114666	c.2605+1G>A	ENST00000287647	NA	splice donor variant	Uncertain	Paternally inherited (het), constitutive in father	Autistic behavior; Delayed speech and language development; Thrombocytosis	DECIPHER v9.26
FANCD2	DDD4K.02005	unknown	3:10136894	c.3974T>G	NM_001018115.1	p.(L1325R)	missense		De novo	developmental Disorder	PMID:28135719
KDM1A	ZH61135	unknown	1:23403725	c.1739A>G	NM_001009999.2	p.(D580G)	missense		De novo	intellectual Disability	PMID:23020937
KDM1A	14329.p1	unknown	1:23409718	c.2420A>G	NM_015013.3	p.(Y807C)	missense		unknown	autism	PMID:28965761
KDM4A	11642.s1	unknown	1:44169774	c.3045A>T	NM_014663.2	p.(K1015N)	missense		De novo constitutive (het)	Control	PMID:25363768
L3MBTL1	11962.p1	unknown	20:42169759	c.2514T>G	NM_032107.4	p.(S838R)	missense		De novo	autism	PMID:25363768
NSD1	AU3646301	unknown	5:176562845	c.741C>A	NM_022455.4	p.(S247R)	missense		De novo	autism	PMID:28263302
NSD1	SC_CHD_FY35816768	unknown	5:176631212	c.1156del1	NM_022455.4	p.(F386Lfs*33)	coding Complex		unknown	congenital heart disease	PMID:27479907
NSD1	1-0184-003	unknown	5:176636718	c.511C>T	NM_172349.2	p.(R171*)	stop-gained		De novo	autism	PMID:28263302
NSD1	DDD4K.02740	unknown	5:176637231	c.1024C>T	NM_172349.2	p.(R342*)	stop-gained		unknown	developmental Disorder	PMID:28135719
NSD1	DDD4K.04231	unknown	5:176637239	c.1033_1034del2	NM_172349.2	p.(W345Vfs*14)	coding Complex		unknown	developmental Disorder	PMID:28135719
NSD1	DDD4K.00253	unknown	5:176637498	c.1292_1293del2	NM_172349.2	p.(R431Qfs*5)	coding Complex		De novo	developmental Disorder	PMID:28135719

NSD1	DDD4K.02019	unknown	5:176638504	c.2297C>G	NM_172349.2	p.(S766*)	stop-gained	unknown	developmental Disorder	PMID:28135719
NSD1	4050-1	unknown	5:176638534	c.2327G>A	NM_172349.2	p.(R776H)	missense	De novo	schizophrenia	PMID:24463507
NSD1	DDD4K.04058	unknown	5:176665274	c.3151C>T	NM_172349.2	p.(R1051*)	stop-gained	unknown	developmental Disorder	PMID:28135719
NSD1	DDD4K.04228	unknown	5:176673677	c.3572-2A>G	NM_172349.2	NA	splice_acceptor_variant	De novo	developmental Disorder	PMID:28135719
NSD1	NDAR_INVJP219FCV_wes1	unknown	5:176684041	c.4048T>C	NM_172349.2	p.(C1350R)	missense	unknown	autism	PMID:25363760
NSD1	DDD4K.00723	unknown	5:176694715	c.4492_4493insA	NM_172349.2	p.(Y1498*fs*1)	coding Complex	unknown	developmental Disorder	PMID:28135719
NSD1	DDD4K.00901	unknown	5:176709563	c.5990A>G	NM_022455.4	p.(Y1997C)	missense	unknown	developmental Disorder	PMID:28135719
NSD1	SC_CHD_FY35816386	unknown	5:176709563	c.5990A>G	NM_022455.4	p.(Y1997C)	missense	unknown	congenital heart disease	PMID:27479907
NSD1	13053.s1	unknown	5:176715871	c.6203C>G	NM_022455.4	p.(T2068S)	missense	De novo	control	PMID:25363768
NSD1	Proband-1498	unknown	5:176719067	c.5564G>A	NM_172349.2	p.(C1855Y)	missense	unknown	mixed	PMID:28959963
NSD1	DDD4K.02982	unknown	5:176720974	c.6605G>A	NM_022455.4	p.(C2202Y)	missense	De novo	developmental Disorder	PMID:28135719
NSD1	DDD4K.00154	unknown	5:176721015	c.6646G>A	NM_022455.4	p.(G2216R)	missense	unknown	developmental Disorder	PMID:28135719
NSD1	1-02563	unknown	5:176722213	c.7845_7849del5	NM_022455.4	p.(K2615Nfs*66)	frameshift	De novo	congenital heart disease	PMID:26785492
NSD2	DDD4K.02817	unknown	4:1918602	c.766_767del2	NM_001042424.2	p.(K257Efs*12)	coding Complex	De novo	developmental Disorder	PMID:28135719
NSD2	AC01-1002-01	unknown	4:1920309	c.1370del1	NM_133335.3	p.(A457Dfs*16)	frameshift	unknown	autism	PMID:25363760
NSD2	DDD4K.02041	unknown	4:1932428	c.1486G>T	NM_133331.2	p.(E496*)	stop-gained	De novo	developmental Disorder	PMID:28135719
NSD2	1-00290	unknown	4:1940175	c.1675-2_1676del4	NM_133330.2	NA	frameshift-near-splice	unknown	congenital heart disease	PMID:26785492
NSD2	Lelieveld_231	unknown	4:1976627	c.3410C>T	NM_001042424.2	p.(S1137F)	missense	De novo	intellectual Disability	PMID:27479843
NSD2	DDD4K.01422	unknown	4:1977033	c.3528_3529del2	NM_001042424.2	p.(F1177*fs*1)	coding Complex	De novo	developmental Disorder	PMID:28135719

NSD2	AU021204	unknown	4:1980530	c.3992C>T	NM_001042424.2	p.(A1331V)	missense		unknown	autism	PMID:28263302
NSD3	ND32630	unknown	8:38187086	c.1391C>T	NM_017778.2	p.(P464L)	missense		De novo	epilepsy	PMID:23934111
PHF8	274736	46XY	X:54013549	c.2065C>T	ENST00000357988	p.Arg689Ter	stop-gained		De novo constitutive (hemi)	Attention deficit hyperactivity disorder; Broad nasal tip; Cleft palate; Delayed speech and language development; Facial asymmetry; Global developmental delay; Hearing impairment; Micrognathia; Seizures; Unilateral cleft lip; Unilateral ptosis; Visual impairment	DECIPHER v9.26
PHF8	359780	46XY	X:54014379	c.1839-2A>G	ENST00000357988	NA	splice acceptor variant	Uncertain	Maternally inherited (hemi), constitutive in mother	Abnormality of the nervous system	DECIPHER v9.26
PHF8	322628	46XY	X:54014379	c.1839-2A>G	ENST00000357988	NA	splice acceptor variant	Likely pathogenic	Maternally inherited (hemi), constitutive in mother	Abnormality of the nervous system	DECIPHER v9.26
PHF8	301931	46XY	X:54022137	c.1420C>T	ENST00000357988	p.Arg474Cys	missense		De novo constitutive (hemi)	Bilateral talipes equinovarus; Frontal upsweep of hair; Functional abnormality of the bladder; Hypospadias; Kyphoscoliosis; Plagiocephaly	DECIPHER v9.26
PHF8	304066	46XX	X:54037638	c.971A>G	ENST00000357988	p.Gln324Arg	missense	Uncertain	De novo constitutive (het)	Abnormal CNS myelination; Abnormality of forebrain morphology; Congenital microcephaly; Febrile seizures; Micrognathia; Prominent nose	DECIPHER v9.26
PHF8	13916.s1	unknown	X:54037669	c.940C>T	NM_001184896.1	p.(L314F)	missense		De novo	Control	PMID:25363768

PHF8	260452	46XY	X:54040854	c.738_739 insT	ENST00000322659	p.His247SerfsTer3	frameshift	Likely pathogenic	Maternally inherited (hemi), constitutive in mother	Attention deficit hyperactivity disorder; Delayed speech and language development; Global developmental delay; Hypermetropia; Lower limb hyperreflexia; Microcephaly; Repetitive compulsive behavior; Tip-toe gait	DECIPHER v9.26
PHF8	274085	46XY	X:54043027	c.704+1 G>A	ENST00000357988	NA	splice donor variant	Pathogenic	De novo constitutive (hemi)	Abnormal size of the palpebral fissures; Abnormality of the outer ear; Autistic behavior; Broad hallux; Broad thumb; Broad-based gait; Coarse facial features; Hypertelorism; Recurrent hand flapping; Severe global developmental delay; Sleep disturbance; Stereotypy; Upslanted palpebral fissure	DECIPHER v9.26
TP53BP1	08C78257	unknown	15:43705517	c.5105G>A	NM_001141980.1	p.(G1702E)	missense		unknown	autism	PMID:25363760
TP53BP1	DDD4K.01006	unknown	15:43748186	c.2605A>G	NM_005657.2	p.(M869V)	missense		unknown	developmental Disorder	PMID:28135719

Variants in red are considered intolerant based on control data (Exome Aggregation Consortium; http://exac.broadinstitute.org/).

Table S2. CNVs in KMT genes from DECIPHER v9.26.

Gene	Patient	Sex	CNV Size	Pathogenicity	Genotype / Class	Inheritance	Phenotypes
KMT5A	285997	46XY	101.30 Mb	Pathogenic	Heterozygous Deletion	De novo constitutive	Abnormal facial shape; Global developmental delay
KMT5A	270716	46XX	2.95 Mb		Heterozygous Deletion	De novo constitutive	Brachydactyly; Cone-shaped epiphysis; Fine hair; Prominent nose; Proportionate short stature; Sparse hair
KMT5A	267744	46XY	3.19 Mb		Heterozygous Deletion	De novo constitutive	Moderate global developmental delay; Short stature; Tracheomalacia
KMT5A	294371	46XX	2.14 Mb	Likely pathogenic	Heterozygous Deletion	De novo constitutive	Intellectual disability; Seizures
KMT5A	306703	46XY	12.20 Mb	Pathogenic	Heterozygous Duplication	Unknown	Cryptorchidism; Hypoparathyroidism; Hypospadias; Iris coloboma; Mixed hearing impairment; Vertebral clefting; Vesicoureteral reflux
KMT5A	274926	46XY	29.81 Mb		Heterozygous Duplication	De novo constitutive	Morphological abnormality of the central nervous system
KMT5A	259576	46XY	386.53 kb		Heterozygous Duplication	Inherited from normal parent	
KMT5A	331326	46XX	281.46 kb	Likely pathogenic	Heterozygous Duplication	Maternally inherited, constitutive in mother	Short stature
KMT5A	256734	46XX	15.76 Mb		Heterozygous Duplication	Imbalance arising from a balanced parental rearrangement	
KMT5A	332457	46XX	92.32 kb	Uncertain	Heterozygous Duplication	Unknown	Abnormal facial shape; Acanthosis nigricans; Delayed speech and language development; Intellectual disability, mild
KMT5A	290464	46XX	15.01 Mb		Heterozygous Duplication	Unknown	
KMT5A	333233	46XY	15.03 Mb		Heterozygous Duplication	Unknown	Hypoplasia of the corpus callosum; Moderate global developmental delay; Neonatal hypoglycemia; Trigonocephaly
KMT5A	283821	46XX	14.04 Mb		Heterozygous Duplication	Unknown	
KMT5A	338407	unknown	307.27 kb	Uncertain	Heterozygous Duplication	Paternally inherited, constitutive in father	Intellectual disability
KMT5A	304366	46XX	449.58 kb	Uncertain	Heterozygous Duplication	Unknown	
KMT5A	370352	46XX	18.71 Mb	Pathogenic	Heterozygous Duplication	De novo constitutive	
KMT5A	250892	46XX	930.49 kb		Heterozygous Triplication	Inherited from parent with similar phenotype to child	Autism; Intellectual disability; Microcephaly; Muscular hypotonia
KMT5B	286222	46XY	134.05 Mb	Pathogenic	Heterozygous Deletion	De novo mosaic	Abnormality of the foot; Abnormality of the hand
KMT5B	257438	46XY	1.29 Mb		Heterozygous Deletion	De novo constitutive	Autism; Intellectual disability
KMT5B	339957	unknown	2.50 Mb	Uncertain	Heterozygous Deletion	De novo constitutive	Autism; Head-banging; Hearing impairment; Seizures; Self-injurious behavior; Severe global developmental delay; Sleep disturbance; Stereotypical body rocking
KMT5B	280831	46XX	5.43 Mb		Heterozygous Deletion	De novo constitutive	

KMT5B	251970	46XX	5.14 Mb		Heterozygous Deletion	De novo constitutive	Aphasia; Cleft palate; Clinodactyly of the 5th finger; Deeply set eye; Feeding difficulties in infancy; Frontal bossing; Hypertelorism; Intellectual disability; Micrognathia; Muscular hypotonia; Patent ductus arteriosus; Short stature; Small for gestational age
KMT5B	251808	46XY	399.01 kb		Heterozygous Deletion	De novo constitutive	Delayed speech and language development; Downslanted palpebral fissures; Intellectual disability; Low-set ears; Macrocephaly; Triangular face
KMT5B	300792	46XX	73.46 Mb	Pathogenic	Heterozygous Duplication	Unknown	Asthma; Delayed fine motor development; Delayed speech and language development; Epicanthus; Global developmental delay; Hypertelorism; Long philtrum; Microtia; Thin upper lip vermilion
KMT5B	280369	46XY	9.37 Mb	Pathogenic	Heterozygous Duplication	De novo constitutive	Cutis laxa; Intellectual disability, moderate; Joint laxity
KMT5B	254643	46XX	6.69 Mb		Heterozygous Duplication	De novo constitutive	Coarse facial features; Hoarse voice; Intellectual disability; Muscular hypotonia; Synophrys
KMT5B	333571	46XY	9.81 Mb	Uncertain	Heterozygous Duplication	De novo mosaic	Bilateral ptosis; Hip dysplasia; Inguinal hernia; Joint hypermobility; Long face; Mitral valve prolapse; Moderate global developmental delay; Pes planus; Prominent forehead; Relative macrocephaly
KMT5B	250851	46XY	7.28 Mb		Heterozygous Triplication	De novo constitutive	Hydrocephalus; Hypoplasia of the corpus callosum; Intellectual disability; Plagiocephaly; Prominent metopic ridge
KMT5C	257434	46XX	399.78 kb		Heterozygous Deletion	Inherited from normal parent	Atrial septal defect; Hypothyroidism; Intellectual disability
KMT5C	252782	other	4.16 Mb		Heterozygous Deletion	Unknown	
KMT5C	300110	unknown	664.05 kb		Heterozygous Deletion	De novo constitutive	Global developmental delay
KMT5C	362540	46XX	9.35 Mb	Pathogenic	Heterozygous Duplication	Imbalance arising from a balanced parental rearrangement	Periventricular gray matter heterotopia
KMT5C	338729	46XY	399.10 kb	Uncertain	Heterozygous Duplication	Unknown	Autistic behavior; Delayed speech and language development
KMT5C	280491	46XY	5.48 Mb		Heterozygous Duplication	De novo constitutive	Global developmental delay
KMT5C	356333	46XY	5.09 Mb	Likely pathogenic	Heterozygous Duplication	Unknown	Intellectual disability; Seizures; Short stature; Specific learning disability
KMT5C	2361	46XY	7.69 Mb		Heterozygous Duplication	Unknown	Intellectual disability; Non-midline cleft lip; Short foot; Short palm; Short stature
KMT5C	306295	unknown	3.69 Mb	Uncertain	Heterozygous Duplication	Unknown	
KMT5C	252490	46XY	4.89 Mb		Heterozygous Duplication	Unknown	Autism; Cognitive impairment
KMT5C	270960	46XX	13.15 Mb		Heterozygous Duplication	Imbalance arising from a balanced parental rearrangement	
KMT5C	275388	46XY	58.83 Mb		Heterozygous Duplication	Unknown	

KMT5C	275426	46XX	7.78 Mb		Heterozygous Duplication	De novo constitutive	Generalized-onset seizure; Global developmental delay; Hypertelorism; Malar flattening; Neonatal hypotonia; Proportionate short stature; Seizures
KMT5C	276191	47XYY	2.81 Mb		Heterozygous Duplication	Paternally inherited, mosaic in father	Intellectual disability, moderate
KMT5C	285720	46XY	8.44 Mb		Heterozygous Duplication	De novo constitutive	Broad forehead; Global developmental delay; Intellectual disability; Motor delay; Strabismus
KMT5C	274515	46XX	5.91 Mb		Heterozygous Duplication	Unknown	Hypodysplasia of the corpus callosum; Mild global developmental delay; Noncommunicating hydrocephalus
KMT5C	280067	46XX	7.80 Mb		Heterozygous Duplication	De novo constitutive	
KMT5C	282304	46XX	2.11 Mb		Heterozygous Duplication	Unknown	Abnormal facial shape; Microcephaly; Severe global developmental delay
KMT5C	274058	46XX	10.63 Mb		Heterozygous Duplication	De novo constitutive	Aggressive behavior; Aplasia/Hypoplasia of the earlobes; Clinodactyly of the 5th finger; Clubbing; Delayed speech and language development; Edema of the dorsum of hands; Generalized tonic seizures; Hypertelorism; Intellectual disability; Long philtrum; Low-set ears; Nephrolithiasis; Round face; Short neck; Short palm; Short stature; Spina bifida occulta; Thickened calvaria; Thoracolumbar scoliosis
KMT5C	292739	46XY	3.89 Mb	Likely pathogenic	Heterozygous Duplication	Unknown	Facial asymmetry; Increased facial adipose tissue; Telangiectasia
KMT5C	339071	46XY	887.86 kb	Uncertain	Heterozygous Duplication	Unknown	
KMT5C	273079	46XY	4.12 Mb		Heterozygous Duplication	De novo constitutive	Delayed gross motor development; Delayed speech and language development; Febrile seizures; Joint laxity; Muscle weakness
KMT5C	296472	46XY	3.54 Mb	Likely pathogenic	Heterozygous Duplication	De novo constitutive	Subcutaneous lipoma; Telangiectases of the cheeks; Telangiectasia

Red=deletions; Blue=duplications; Green=triplications.

Table S3. SNVs in KMT genes.

Gene	Patient	Sex	gDNA Position (GRCh37)	cDNA Change	Transcript	Protein Change	Consequence	Pathogenicity	Inheritance / Genotype	Phenotypes	Source
KMT 5B	ClinVar:235894	unknown	11:67925316	c.2497G>T	NM_017635	(p.Glu833Ter)		Likely pathogenic (Last reviewed: May 12, 2016)	Unknown		ClinVar
KMT 5B	ClinVar:521155	unknown	11:67925651	c.2162G>A	NM_017635	(p.Arg721His)		Uncertain significance (Last reviewed: Jul 5, 2016)	Unknown	Inborn genetic diseases	ClinVar
KMT 5B	ClinVar:446522	unknown	11:67926255 - 67926256	c.1557_1558del	NM_017635	(p.Asn520Serfs)		Pathogenic (Last reviewed: Dec 5, 2017)	Unknown	Mild to moderate intellectual disability, autism spectrum disorder, +2 SD on height, MRI of the brain showed enlarged perivascular areas, unilateral cryptorchidism	ClinVar
KMT 5B	ClinVar:521217	unknown	11:67938489	c.970T>G	NM_017635	(p.Cys324Gly)	missense	Likely pathogenic (Last reviewed: Sep 6, 2016)	Unknown	Inborn genetic diseases	ClinVar
KMT 5B	ClinVar:374236	unknown	11:67938529	c.930del	NM_017635	(p.Phe311Serfs)	frameshift	Uncertain significance	Unknown	Language retardation	ClinVar
KMT 5B	ClinVar:446523	unknown	11:67939039	c.791G>C	NM_017635	(p.Trp264Ser)	missense	Pathogenic (Last reviewed: Dec 5, 2017)	Unknown	MENTAL RETARDATION, AUTOSOMAL DOMINANT 51	ClinVar

KMT 5B	ClinVar:446521	unknown	11:67939105	c.725del	NM_017635	(p.Leu242Hisfs)	frameshift	Pathogenic (Last reviewed: Dec 5, 2017)	Unknown	Mild to moderate intellectual disability, +1 SD on height, febrile seizures, symptoms of attention deficit disorder, but no formal diagnosis, EEG has shown mild epileptic abnormalities; CT and MRI show wide ventricles	ClinVar
KMT 5B	ClinVar:521728	unknown	11:67939172	c.658C>T	NM_017635	(p.Arg220Ter)		Pathogenic (Last reviewed: May 8, 2017)	Unknown	Inborn genetic diseases	ClinVar
KMT 5B	ClinVar:560606	unknown	11:67941365	c.559C>T	NM_017635	(p.Arg187Ter)		Pathogenic (Last reviewed: Sep 10, 2018)	Unknown	MENTAL RETARDATION, AUTOSOMAL DOMINANT 51	ClinVar
KMT 5B	ClinVar:559640	unknown	11:67953301	c.255del	NM_017635	(p.Ser86Valfs)	frameshift	Pathogenic (Last reviewed: Mar 28, 2018)	Unknown	MENTAL RETARDATION, AUTOSOMAL DOMINANT 51	ClinVar
KMT 5B	ClinVar:560605	unknown	11:67953337	c.219delC	NM_017635	(p.Ala74Profs)		Pathogenic (Last reviewed: Sep 10, 2018)	Unknown	MENTAL RETARDATION, AUTOSOMAL DOMINANT 51	ClinVar
KMT 5A	ClinVar:161732	unknown	12:123879591	c.290-3C>A	NM_020382			Uncertain significance	Unknown	Malignant tumor of prostate	ClinVar
KMT 5B	293182	46XX	11:67925207	c.2606T>C	NM_017635	p.Ile869Thr	missense		De novo constitutive (het)	Atrial septal defect; Global developmental delay; Hip	DECIPHER v9.26

										dislocation; Short stature; Stridor	
KMT 5B	266454	46XX	11:679262 76	c.1537G>A	NM_017635	p.Ala513Thr	missense	Uncertain	Maternally inherited (het), constitutive in mother	Dystonia; Hypertonia; Motor delay; Nystagmus	DECIPHER v9.26
KMT 5B	307467	46XY	11:679266 30	c.1183C>T	NM_017635	p.Arg395Ter	stop-gained		De novo constitutive (het)	Delayed speech and language development; Hypermetropia; Joint hypermobility; Moderate global developmental delay	DECIPHER v9.26
KMT 5B	340407	46XX	11:679345 50	c.1073T>C	NM_017635	p.Leu358Ser	missense	Uncertain	De novo constitutive (het)	Abnormality of the nervous system	DECIPHER v9.26
KMT 5B	300235	46XX	11:679391 72	c.658C>T	NM_017635	p.Arg220Ter	stop-gained		De novo constitutive (het)	Abnormal aortic valve morphology; Abnormal emotion/affect behavior; Aortic regurgitation; Aortic root aneurysm; Arachnodactyly; Asymmetry of the thorax; High palate; Mild global developmental delay; Pes planus; Shyness; Thoracic scoliosis	DECIPHER v9.26

KMT 5B	368213	46XX	11:67941315	c.608_609 insA	NM_017635	p.Tyr203Ter	stop-gained	Likely pathogenic	De novo constitutive (het)	Growth abnormality	DECIPHER v9.26
KMT 5B	304745	46XY	11:67941343	c.581G>A	NM_017635	p.Gly194Glu	missense	Likely pathogenic	De novo constitutive (het)	Arnold-Chiari malformation; Autistic behavior; Cerebellar malformation; Hyperextensibility of the finger joints; Intellectual disability, moderate; Polyphagia; Strabismus	DECIPHER v9.26
KMT 5B	265236	46XY	11:67941365	c.559C>T	NM_017635	p.Arg187Ter	stop-gained		De novo constitutive (het)	Abnormal facial shape; Delayed speech and language development; Frontal bossing; Generalized hypotonia; Inverted nipples; Microtia; Proptosis; Seizures; Specific learning disability	DECIPHER v9.26
KMT 5B	305032	46XX	11:67942486	c.542A>G	NM_017635	p.His181Arg	missense		De novo constitutive (het)	Arnold-Chiari type I malformation; Moderate global developmental delay	DECIPHER v9.26

KMT5B	277906	46XX	11:67953336-67953337	c.219 del	NM_017635	p.Ala74ProfsTer10	frameshift		De novo constitutive (het)	Autism; Broad forehead; Delayed gross motor development; Delayed speech and language development; Epicanthus; Facial asymmetry; Finger joint hypermobility; Global developmental delay; High palate; Hypermobility of distal interphalangeal joints; Intellectual disability; Large forehead; Macrocephaly; Overgrowth; Posteriorly rotated ears	DECIPHER v9.26
KMT5B	NDAR_INVNE346GDX_wes1	unknown	11:67925466	c.2347C>T	NM_017635	p.(R783*)	stop-gained		Unknown	Autism spectrum disorder	PMID:25363760
KMT5B	DEASD_0109_001	unknown	11:67941367-67941370	c.554_557del4	NM_016028	p.(Y185Cfs*27)	frameshift		Unknown	Autism spectrum disorder	PMID:25363760
KMT5B	12864.p1	unknown	11:67938481	c.977+1G>A	NM_017635	NA	splice-donor		De novo constitutive (het)	Autism spectrum disorder, Intellectual disability, low average	PMID:25363768

										receptive vocabulary, History of elevated attention and withdrawal problems, history of sleep problems	
KMT5B	11519.p1	unknown	11:67939039	c.791G>C	NM_017635	p.(W264S)	missense		De novo constitutive (het)	Autism spectrum disorder, moderate intelelctual disability, Low receptive and expressive vocabularies; Speech Sound Disorder, history of gastrointestinal dysfunction, agression towards other peers, combined variable immune deficiency, sleep problems.	PMID:25363768
KMT5B	1-06015	unknown	11:67926495	c.1318A>C	NM_017635	p.(K440Q)	missense		Unknown	Congenital heart disease	PMID:26785492
KMT5B	1-01151	unknown	11:67942601	c.427C>T	NM_017635	p.(R143C)	missense		Unknown	Congenital heart disease	PMID:26785492
KMT5B	Lelieveld_146	unknown	11:67953389	c.166_167insAATG	NM_016028	p.(G56Efs*3)	frameshift		Unknown	Intellectual disability	PMID:27479843
KMT5B	1-0466-003	unknown	11:67925217	c.2596T>C	NM_017635	p.(S866P)	missense		De novo constitutive (het)	Autism spectrum disorder	PMID:28263302

KMT5B	AU000704	unknown	11:67926065-67926140	c.1673_1748del76	NM_017635	p.(P558Lfs*38)	frameshift		De novo constitutive (het)	Autism spectrum disorder	PMID:28263302
KMT5A	AU002406	unknown	12:123888130	c.608G>A	NM_020382	p.(R203K)	missense		Unknown	Autism spectrum disorder	PMID:28263302
KMT5B	Proband-1	unknown	11:67939049-67939050	c.780_781del2	NM_017635	p.(A261Sfs*13)	frameshift		Unknown	Mixed	PMID:28959963
KMT5B	11729.p1	unknown	11:67926275	c.1538C>T	NM_017635	p.(A513V)	missense		De novo constitutive (het)	Autism spectrum disorder, intellectual disability, cognitive and adaptive abilities extremely impaired, febrile seizures, attention and affective problems, Tourette’s/Tics diagnosis improved with fever; history of significant sleep problems	PMID:28965761
KMT5B	12859.p1	unknown	11:67933707	c.*734C>T	NM_016028	NA	3-prime-UTR		De novo constitutive (het)	Autism spectrum disorder	PMID:28965761
KMT5B	Leiden_D1.12.00933	unknown				p.Arg540Gln	missense		Maternal	Intellectual disability/developmental delay, motor delay, bilateral epicanthal folds	
KMT5B	Swedish_1720-08D	unknown				p.Asn389Lysfs*6	frameshift		Unknown	Intellectual disability,	

Autism spectrum disorder, no language, delayed psychomotor development, +2 SD height, febrile in infancy, unilateral pes equinovarus and bilateral pes plano valgus; EEG showed slow activity

Shaded cells may be duplicate entries into multiple databases.

Table S4. Genetic variation tolerance scores from the Exome Aggregation Consortium by gene.

Gene (HUGO)	Alias	H4K20 Function	Synonymous (z)	Missense (z)	LoF (pLI)	Associated Disorder
*KMT5A**	*SETD8*	Writer	0.66	2.44	0.95	-
*KMT5B**	*SUV420H1*	Writer	-0.29	2.71	1.00	MR, AD
*KMT5C**	*SUV420H2*	Writer	-1.82	1.99	0.69	-
NSD1	*SOTOS*	Writer	-0.73	2.38	1.00	Sotos syndrome
NSD2	*WHSC1, MMSET*	Writer	-0.76	4.1	1.00	
NSD3	*WHSC1L1*	Writer	0.27	3.54	1.00	
KDM4A	*JMJD2A*	Eraser	0.53	3.59	1.00	-
PHF8		Eraser	-0.38	4.59	1.00	MR, XLR
KDM1A	*LSD1*	Eraser	0.95	5.56	0.99	Cleft palate, psychomotor retardation, and distinctive facial features, AD
L3MBTL1		Reader	0.26	0.08	0.00	-
TP53BP1		Reader	0.13	0.67	1.00	-
FANCD2		Reader	0.89	-0.25	0.00	Fanconi anemia, AR

Shaded cells indicate genetic intolerance based on control data (Exome Aggregation Consortium; http://exac.broadinstitute.org/); LoF=loss of function. * Gene information is redundant to Table 1 in the main text but provided here for clarity.

Table S5. Phenotypic summary of available data for KMT gene variant carriers.

Phenotype	*KMT5A*		*KMT5B*		*KMT5C*	
	CNV	SNV	CNV	SNV	CNV	SNV
Intellectual disability	3		6	11	5	
Global developmental delay			3	5	4	
Delayed speech and language development	2		2	7	3	
Short stature	2		1	1	3	
Tall stature/overgrowth/growth abnormality				5		
Hypertelorism			2		2	
Seizures			1	4	2	
Short palm					2	
Telangiectasia					2	
High palate			1	2		
Muscular hypotonia			2	2		
Motor delay			1	5		
Abnormal facial shape	2		1			
Autism spectrum disorder		1	2	13		
